# Investigating human-derived lactic acid bacteria for alcohol resistance

**DOI:** 10.1186/s12934-024-02375-4

**Published:** 2024-04-24

**Authors:** Sini Kang, Jing Long, Myeong Soo Park, Geun Eog Ji, Ying Ju, Seockmo Ku

**Affiliations:** 1 Cooperative Innovation Center of Industrial Fermentation (Ministry of Education & Hubei Province), Key Laboratory of Fermentation Engineering (Ministry of Education), National “111” Center for Cellular Regulation and Molecular Pharmaceutics, Hubei Key Laboratory of Industrial Microbiology, School of Life Sciences and Health, Hubei University of Technology, Wuhan, Hubei 430068 China; 2 Department of Food and Nutrition, Research Institute of Human Ecology, Seoul National University, Seoul, 08826 South Korea; 3 Research Center, BIFIDO Co., Ltd, Hongcheon, 25117 South Korea; 4 Department of Food Science and Technology, Texas A&M University, College Station, TX 77843 USA

**Keywords:** Ethanol tolerance, Lactic acid bacteria, Adhesion ability, Whole genome sequencing

## Abstract

**Background:**

Excessive alcohol consumption has been consistently linked to serious adverse health effects, particularly affecting the liver. One natural defense against the detrimental impacts of alcohol is provided by alcohol dehydrogenase (ADH) and acetaldehyde dehydrogenase (ALDH), which detoxify harmful alcohol metabolites. Recent studies have shown that certain probiotic strains, notably *Lactobacillus* spp., possess alcohol resistance and can produce these critical enzymes. Incorporating these probiotics into alcoholic beverages represents a pioneering approach that can potentially mitigate the negative health effects of alcohol while meeting evolving consumer preferences for functional and health-centric products.

**Results:**

Five lactic acid bacteria (LAB) isolates were identified: *Lactobacillus paracasei* Alc1, *Lacticaseibacillus rhamnosus* AA, *Pediococcus acidilactici* Alc3, *Lactobacillus paracasei* Alc4, and *Pediococcus acidilactici* Alc5. Assessment of their alcohol tolerance, safety, adhesion ability, and immunomodulatory effects identified *L. rhamnosus* AA as the most promising alcohol-tolerant probiotic strain. This strain also showed high production of ADH and ALDH. Whole genome sequencing analysis revealed that the *L. rhamnosus* AA genome contained both the *adh* (encoding for ADH) and the *adhE* (encoding for ALDH) genes.

**Conclusions:**

*L. rhamnosus* AA, a novel probiotic candidate, showed notable alcohol resistance and the capability to produce enzymes essential for alcohol metabolism. This strain is a highly promising candidate for integration into commercial alcoholic beverages upon completion of comprehensive safety and functionality evaluations.

**Supplementary Information:**

The online version contains supplementary material available at 10.1186/s12934-024-02375-4.

## Introduction

The consumption of alcohol, especially in excess, has been associated with significant adverse health risks. Over time, alcohol abuse can cause severe health issues, most notably to the liver [1]. Alcoholic liver disease (ALD) emerges when the liver becomes inflamed and damaged due to prolonged exposure to alcohol [2].

The body’s natural defense against the harmful effects of alcohol relies on two critical enzymes. Alcohol dehydrogenases (ADH) convert ethanol into acetaldehyde, a toxic intermediary. This acetaldehyde is subsequently transformed by aldehyde dehydrogenases (ALDH) into acetic acid, a less harmful and easily excreted metabolite [3, 4]. Recent studies have spotlighted the alcohol resistance of specific strains of probiotics, especially *Lactobacillus*, which can produce both ADH and ALDH enzymes [5]. These unique characteristics indicates potential therapeutic applications in reducing the adverse effects of excessive alcohol consumption.

For decades, various organizations have striven to reduce alcohol consumption, yet the outcomes have been largely marginal [6]. Given this backdrop, we deliberated on the possibility of adopting a different approach in the alcoholic beverage domain. In Western cultures, major alcoholic beverages, like beer and whiskey, are processed using methods including filtering or distillation, but these processes leave few living microorganisms in the beverage [7, 8]. Most commercially-produced beers also undergo pasteurization, again eliminating most live microbes.

By contrast, makgeolli, a traditional Korean alcoholic drink, employs a distinct fermentation methodology that utilizes both lactic acid bacteria and yeast from the fermentation starter known as “nuruk.” This process results in a co-fermentation that produces both alcohol and lactic acid [9, 10]. Notably, even mass-produced makgeolli emphasizes the preservation of these live microorganisms, with certain brands boasting up to 100 billion lactic acid bacteria per serving [11]. The resurgence of the craft brewing industry and an evolving consumer preference for varied beer flavors have led to a growing popularity of sour beer in the U.S. This type of beer requires lactic acid fermentation to introduce a distinct tartness [12]. Harnessing lactic acid bacteria to provide this tartness would also introduce their inherent alcohol resistance and possible ALD-mitigating properties through ADH and ALDH enzyme production. The reduction in ALD could revolutionize alcoholic beverage development and lead to drinks that have actual health benefits.

This possibility has prompted our group to isolate and characterize various strains of lactic acid bacteria from human feces. We evaluated these strains for their survivability across a spectrum of alcohol concentrations, ranging up to 12% (v/v). We identified the *Lacticaseibacillus rhamnosus* AA strain (*L. rhamnosus* AA) as particularly alcohol resistant, and it showed growth in actual commercial alcoholic beverages. Through whole-genome sequencing, we detected the presence of both the *adh* and *adhE* genes in this strain. Recognizing its potential for applications in food or alcoholic beverages, we undertook a comprehensive biosafety assessment, emphasizing antibiotic resistance, IL-8 production capability, and ammonia production tendencies, with the goal of ascertaining the safety of *L. rhamnosus* AA for human consumption.

## Materials and methods

### Isolation and screening of ethanol-tolerant LAB isolates from human feces

Fresh fecal samples were collected from five children (1–6 years old), following a protocol approved by the Institutional Review Board of Seoul National University (IRB No. 1702/002–013). LAB strains were isolated on *Lactobacillus* Selection (LBS) agar (Difco, Sparks, MD, USA). In total, 318 morphologically different microbial colonies were obtained and subsequently cultured at 37 °C under anaerobic conditions in De Man, Rogosa, and Sharpe (MRS) medium (Becton Dickinson, MD, USA) containing 0.05% L-cysteine hydrochloride. The bacterial stocks were stored at − 80 °C in 17% glycerol (utilized as a cryoprotectant). To screen ethanol-tolerant isolates, the LAB were cultured anaerobically at 37℃ in MRS broth containing 5%, 7%, and 10% (v/v) absolute ethanol (EMSURE®, Darmstadt, Germany). Five strains that grew in 10% ethanol were selected and identified by phylogenetic analysis of their 16 S rRNA gene sequences.

### Ethanol tolerance assessment of the LAB isolates

The screened LAB isolates were cultured in MRS broth containing 0, 2%, 5%, 8%, 10%, 12% (v/v) ethanol for 20 h at 37 °C. Bacterial growth was recorded at OD 600 nm. In addition, 1 × 10^7^ colony-forming units per milliliter (CFU)/mL of the isolates were inoculated into the Korean traditional alcoholic beverages Makgeolli (6% alcohol) and Baekseju (13% alcohol). The beverages were sterilized by filtration through 0.2 μm filters prior to use, and the alcohol concentration of Baekseju was diluted to 6% with sterilized water. The LAB isolates were cultured for 24 h at 37 °C, and their growth were monitored at OD 600 nm. Bacterial counts were measured every 6 h by culturing samples on MRS agar plates under anaerobic conditions. The averages were expressed as CFU/mL of each sample. Survival rate was calculated as follows: survival Rate (%) = (CFU mL^− 1^ of each time sample / CFU mL^− 1^ of sample at 0 h) × 100%.

### Safety evaluation of the LAB isolates

Bacterial ammonia production was determined using the method previously described by Chaney AL and Marbach EP [13]. The selected strains were cultured in brain-heart infusion (BHI) broth at 37℃ for 5 days. The culture supernatants were reacted for 30 min at room temperature with 10 g/L phenol (Junsei Chemical, Japan), 0.05 g/L sodium nitroprusside (Sigma-Aldrich, MO, USA), 5.0 g/L sodium hydroxide (Sigma-Aldrich), and 0.42 g/L sodium hypochlorite (Sumchun, Korea), and the resulting blue color was measured at 625 nm. *Enterobacter cloacae* KCTC 2361 and *Enterococcus faecalis* KCTC 3511 were used as positive controls, and *Bifidobacterium bifidum* BGN4 was used as a negative control [14]. In the hemolytic test, blood agar was prepared by adding 5% horse blood and 1.5% agar to BHI broth, and the bacterial isolates were cultured on the blood agar plate at 37℃ for 2 days. *Listeria ivanovii* subsp. *ivanovii* ATCC 19,119 was used as a positive control [14].

### Cell line preparation

The Caco-2 and HT-29 (KCLB 30,038) cell lines were purchased from Korea Cell Line Bank. The cells were seeded into 24-well plates at a density of 1 × 10^6^ cells per well, and routinely cultured at 37 °C in an atmosphere of 5% CO_2_ in Dulbecco’s Modified Eagle’s Medium (DMEM; Sigma-Aldrich) supplemented with 10% (v/v) heat-inactivated fetal bovine serum (FBS) and 1% penicillin/streptomycin (Sigma Aldrich, USA). The medium was replaced with dimethylsulfoxide (DMSO) without antibiotic when the cells reached 90% confluency.

### Bacterial adhesion to cells

Cultures of the LAB isolates were washed twice with sterile phosphate buffered saline (PBS) and resuspended in DMSO medium. Bacterial pellets (10^8^ CFU/mL) were added to Caco-2 monolayer-containing chambers at a bacteria-to-cell ratio of 100:1. The chamber slides were incubated at 37 °C in an atmosphere of 5% CO_2_ for 1 h. After the incubation, the monolayers were washed three times with sterilized PBS buffer and lysed with 2× trypsin (1 g/L, Sigma-Aldrich) for 10 min at 37 °C in an atmosphere of 5% CO_2_. The DNA was then extracted from the adhering using an MG Cell Genomic DNA Extraction SV kit (Doctor protein, Korea) and measured by quantitative real-time PCR (RT-qPCR) with SYBR® Premix Ex TaqTM Kit (Takara, Tokyo, Japan) and the Step One Plus and Step One (Applied Biosystem, USA). The PCR conditions were 30 s at 95 °C, followed by 40 cycles of denaturation at 95 °C for 5 s and annealing at 60 °C for 34 s, coupled with melt curve analysis. According to previous studies [15, 16], the primers used in this study were displayed in Table S.

The effects of carbon sources on bacterial growth and adhesion to the cell cultures were tested using glucose (glu, a positive control), fructose (fru), galactose (gal), fructooligosaccharides (FOS), butyl-fructooligosaccharides (B-FOS), 2-fucosyllactose (2-FL), sucrose (suc) and lactose (lac). The selected LAB isolates were cultured anaerobically at 37℃ in MRS broth (without glucose) containing 2% (w/v) of the different carbon sources, and the bacterial growth was determined by optical measurements at OD 600 nm. The carbon sources (4.5 g/L) were prepared in DMEM without glucose medium (Gibco® Carlsbad, USA). One mL of each bacterial suspension (10^8^ CFU/mL) in DMEM was added to each Caco-2 monolayer chamber and incubated for 1 h. The adhered bacteria were quantified by RT-qPCR.

### Lipopolysaccharides (LPS)-induced interleukin 8 (IL-8) production

Upon formation of monolayers of Caco-2 cells and HT-29 cells, 100 ng/mL lipopolysaccharides (LPS, Sigma-Aldrich), 10^8^ CFU/mL active bacterial suspension and different carbon sources were added. The samples were cultured for 6 h at 37 °C in an atmosphere of 5% CO_2_/95% air. The culture medium was then centrifuged for 10 min and the supernatant was collected for determination of Interleukin-8 (IL-8) production using a Human IL-8 ELISA set (BD, USA).

### ADH and ALDH activities

The ADH and ALDH activities were tested using an alcohol dehydrogenase activity assay kit and an aldehyde dehydrogenase activity colorimetric assay kit (Sigma-Aldrich). The bacterial suspension was washed twice with PBS to remove the MRS medium and the cell concentration was adjusted to 10^8^ CFU/mL. The cells were extracted by sonication at 28% power (40 kHz) for 5 min.

### Whole genome sequencing (WGS) analysis

The bacterial genomic DNA was extracted using an MG™ Cell Genomic DNA Extraction SV Miniprep (MGmed, Korea), according to the manufacturer’s instructions. The extracted DNA was fragmented with a Nextera transposome and simultaneously tagged with adapter sequences. The tagged DNA was then amplified with index 1 and 2 adapters in the PCR step. After purifying with AMPure XP beads and 80% ethanol, the quantity of dual-indexed sequencing was normalized prior to the pooled library construction. The WGS of the pooled library was analyzed using an Illumina MiSeq sequencer (Illumina, USA). The bioinformatics analysis was conducted with EZBioCloud Apps provided by ChunLab Co., Ltd. (Seoul, Korea). The general genomic information included DNA fragment locations, fragment lengths, and evolutionary genealogy of genes: non-supervised orthologous groups (EggNOG).

### Statistical analysis

Data were analyzed via one-way or two-way analysis of variance (ANOVA) with Duncan’s multiple comparisons test. All experiments were repeated in triplicate. A value of *p* < 0.05 was considered statistically significant. All statistical analyses were performed using Graph-Pad Prism 9.

## Results and discussion

### Screening and selection of ethanol-tolerant LAB strains

Of the 318 bacterial strains initially isolated from human feces, a mere five strains demonstrated growth in the challenging environment of 10% (v/v) ethanol in MRS broth. These select strains were identified through detailed 16 S rRNA analysis and were subsequently designated as *Lactobacillus paracasei* Alc1 (*L. paracasei* Alc1), *L. rhamnosus* AA, *Pediococcus acidilactici* Alc3 (*P. acidilactici* Alc3), *Lactobacillus paracasei* Alc4 (*L. paracasei* Alc4), and *Pediococcus acidilactici* Alc5 (*P. acidilactici* Alc5).

**Fig. 1:**
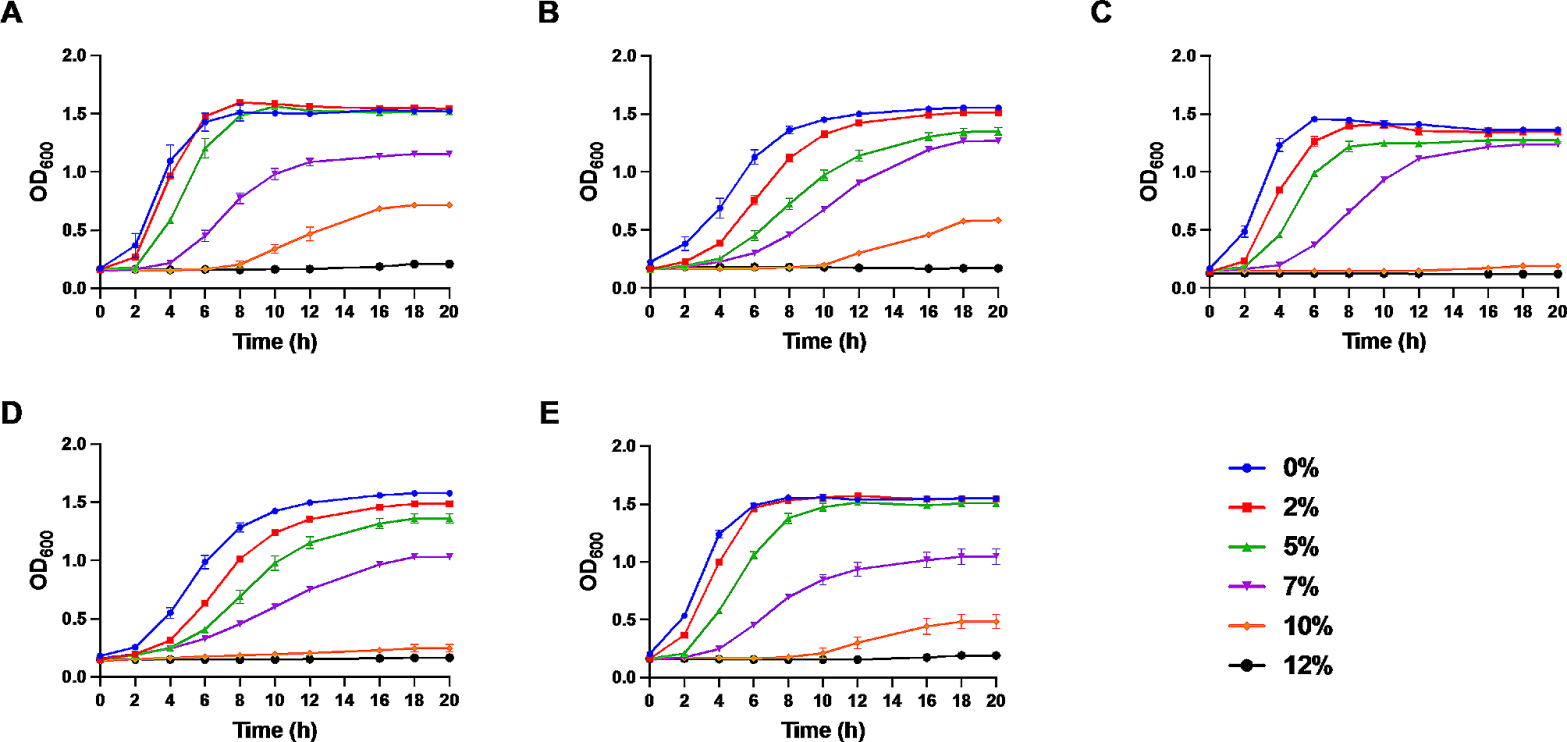
Growth curve of ethanol-tolerant LAB in 0, 2%, 5%, 8%, 10%, 12% (v/v) of ethanol-added MRS broth. (**A**) *L. paracasei* Alc1, (**B**) *L. rhamnosus* AA, (**C**) *P. acidilactici* Alc3, (**D**) *L. paracasei* Alc4, (**E**) *P. acidilactici* Alc5

Figure 1 shows the growth curves for these ethanol-tolerant LAB strains cultivated for 20 h in MRS broth with varying ethanol concentrations ranging from 0 to 12% (v/v). Addition of ethanol prolonged the lag phase of probiotic growth in an ethanol-dependent manner when compared to the control. Specifically, ethanol concentrations up to 5% had minimal inhibitory effects on the bacterial growth, with the OD at 600 nm showing an upward trend after 2 h of cultivation. However, introducing ethanol at 8% ethanol slowed the growth rate, while also delaying the onset of the logarithmic growth phase to 4 h. Only *L. paracasei* Alc1, *L. rhamnosus* AA, and *P. acidilactici* Alc5 were capable of growth in 10% ethanol, and this sluggish growth was observable only after an extended 10 h cultivation period. Importantly, all five strains showed complete growth inhibition at ethanol concentrations of 12% and above.

**Fig. 2:**
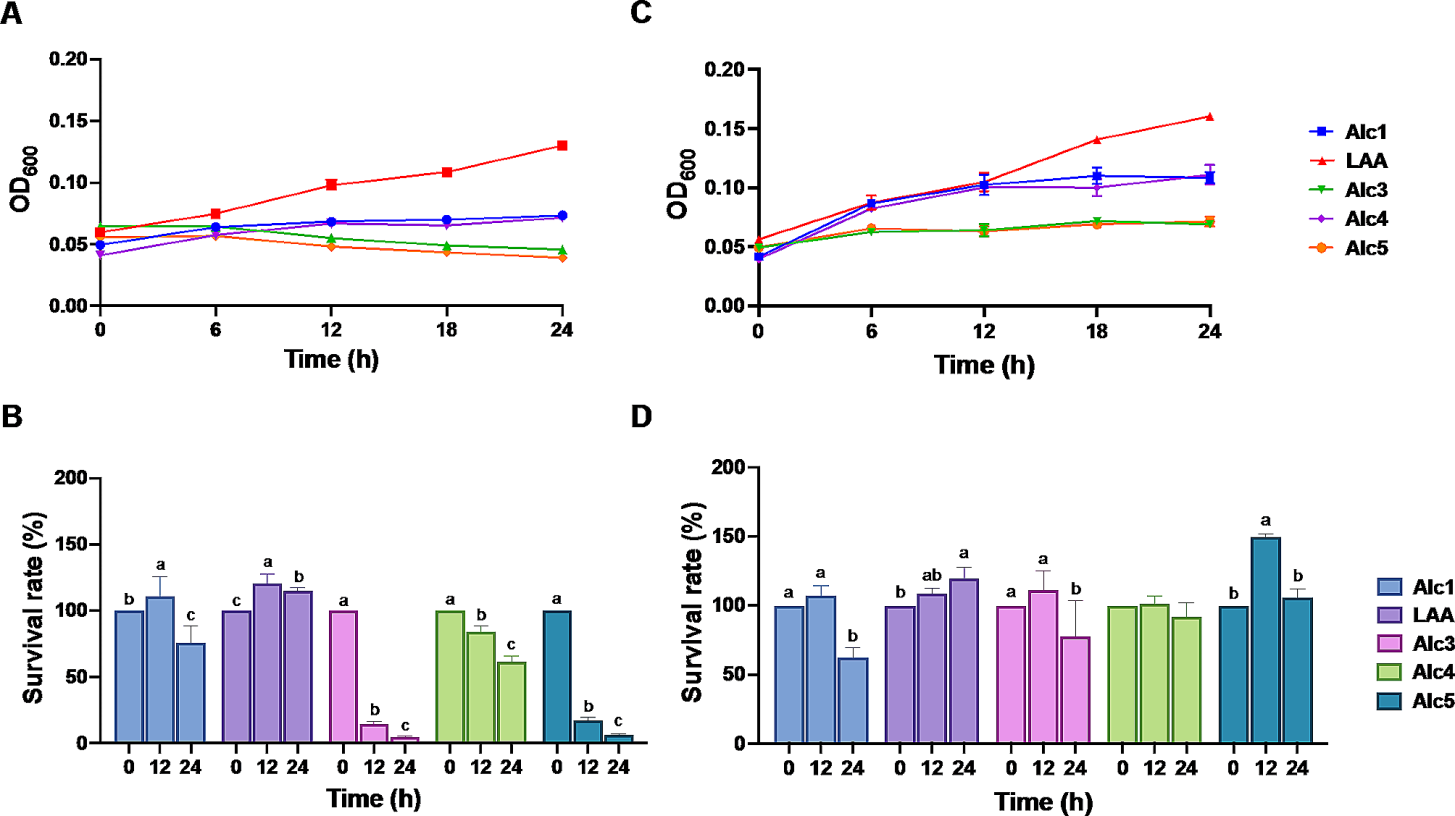
Growth curves (**A**) and survival rates (**B**) of ethanol-tolerant LAB in Makgeolli (**A**, **B**) and Baekseju (**C**, **D**). Alc1, *L. paracasei* Alc1; LAA, *L. rhamnosus* AA; Alc3, *P. acidilactici* Alc3; Alc4, *L. paracasei* Alc4; Alc5, *P. acidilactici* Alc5. Treatments with different letters are significantly different at *p* < 0.05

Figure 2 shows the growth dynamics and survival rates of the five ethanol-tolerant LAB strains in two commercially available Korean alcoholic beverages: makgeolli (A, B) and baekseju (C, D). The growth curves (A, C) demonstrate the evolution of the bacterial populations over time. In makgeolli (A), *L. rhamnosus* AA showed a consistent growth trajectory, maintaining a robust profile throughout the 24 h cultivation period. By contrast, the other two strains showed limited growth, with *P. acidilactici* Alc3 and *P. acidilactici* Alc5 demonstrating notable stagnation, indicating their diminished survival in the makgeolli environment. Conversely, in baekseju (C), *L. rhamnosus* AA emerged as the most successful strain with an impressive growth pattern. By contrast, *L. paracasei* Alc1 and *L. paracasei* Alc4 exhibited moderate growth, whereas the growth of *P. acidilactici* Alc3 and *P. acidilactici* Alc5 appeared to be entirely suppressed, as their growth trajectories flatlined. The bar graphs (B, D) further quantify these observations by representing the survival rates of each strain in both alcoholic environments. In makgeolli (B), the survival rate of *L. rhamnosus* AA indicated successful growth, while survival of the other strains, especially *P. acidilactici* Alc3 and *P. acidilactici* Alc5, was significantly reduced. Similarly, in baekseju (D), the growth of *L. rhamnosus* AA dominated, followed by *L. paracasei* Alc1 and *L. paracasei* Alc4, whereas *P. acidilactici* Alc3 and *P. acidilactici* Alc5 displayed minimal to no survival.

### Safety evaluation

The selected LAB strains were evaluated for consumer safety by measuring ammonia production, hemolytic activity, and antibiotic susceptibility. Ammonia is one of the bacterial fermentation by-products that impair intestinal health [17, 18]. Some LAB with high decarboxylase activities (i.e., tryptophanase) show abnormal amino acid metabolism and excessive ammonia production [19], which can raise the pH and adversely affect the quality of fruit juice, wine, and other beverages by releasing ammonia [20]. In this study, *Enterobacter cloacae* KCTC 2361 and *Enterococcus faecalis* KCTC 3511 were used as positive controls and *Bifidobacterium bifidum* BGN4 was used as a negative control [21]. No ammonia production was observed by any of the five LAB strains (Table 1).

**Table 1:** Bacterial ammonia production

Strain	Ammonia (µg/mL)
*L. paracasei* Alc1 *L. rhamnosus* AA *P. acidilactici* Alc3	negative negative negative
*L. paracasei* Alc4 *P. acidilactici* Alc5 *B. bifidum* BGN4	negative negative negative
*E.* KCTC 2361	11.5 ± 0.9
*E.* KCTC 3511	0.2 ± 0.6

Values are expressed as mean ± SD (*n* = 3). *p* < 0.05

**Fig. 3:**
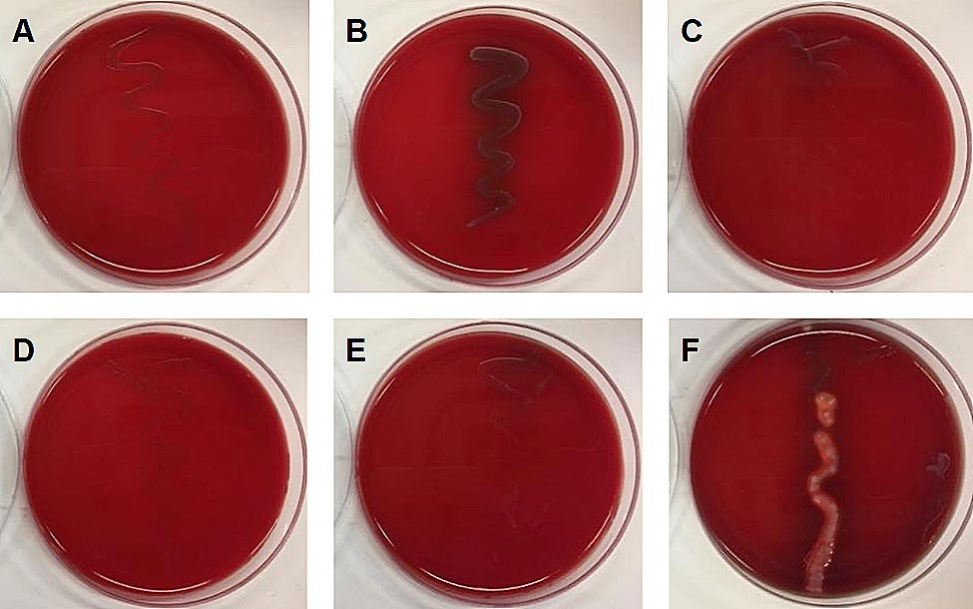
Hemolytic activity test. (**A**) *L. paracasei* Alc1 (**B**) *L. rhamnosus* AA (**C**) *P. acidilactici* Alc3 (**D**) *L. paracasei* Alc4 (**E**) *P. acidilactici* Alc5 (**F**) *Listeria ivanovii* subsp. *ivanovii* ATCC 19,119 (positive control)

Hemolytic activity by pathogens is another common concern for human safety as it can result in anemia and edema and impair gut integrity. The LAB are commensal microbiota, but the risk of hemolysis still needs to be ruled out [21]. *Listeria ivanovii* subsp. *ivanovii* ATCC 19,119 was utilized as a positive control, which was indicated by a color change in the periphery of the colonies. Figure 3 shows that none of the five selected LAB strains displayed any hemolysis activities.

Antibiotic resistance genes in a probiotic genome can be transferred to pathogenic bacteria to generate antibiotic-resistant pathogens [14, 22]. Here, microbiological cut-off values were utilized to distinguish the susceptible strains from strains with acquired resistance according to the distribution of minimum inhibitory concentrations (MICs) of the antibiotics in bacterial populations belonging to a single genus or species [23]. As shown in Table 2, the MIC values for *L. rhamnosus* AA were equal to or lower than the established EFSA cut-off values, indicating that *L. rhamnosus* AA was susceptible to the nine antibiotics tested in this study. The remaining strains were resistant to chloramphenicol. *L. paracasei* Alc 1 was also resistant to kanamycin and tetracycline, and *P. acidilactici* Alc3 and *P. acidilactici* Alc5 were resistant to tetracycline.

**Table 2:** Antimicrobial susceptibility of the LAB isolates

	MIC Value (mg/L)					Microbiological cut-off values
Antibiotics	Alc1	LAA	Alc3	Alc4	Alc5	
Ampicillin	1	1	2	2	2	4
Chloramphenicol	8	4	8	8	8	4
Clindamycin	0.125	0.25	< 0.032	0.125	0.0625	1
Erythromycin	2	2	2	1	2	4
Gentamicin	8	2	4	8	4	16
Kanamycin	128	32	64	64	64	64
Neomycin	32	4	8	16	16	32
Streptomycin	32	8	32	32	32	32^a^, 64^b^
Tetracycline	16	0.25	32	8	32	8

MIC, minimal inhibitory concentrations; n.r.=not required; a, microbiological cut-off value of *Lactobacillus* spp; b, microbiological cut-off value of *Pediococcus* spp; Alc1, *L. paracasei* Alc1; LAA, *L. rhamnosus* AA; Alc3, *P. acidilactici* Alc3; Alc4, *L. paracasei* Alc4; Alc5, *P. acidilactici* Alc5

### Effects of carbon sources on bacterial adhesion and immunology

**Fig. 4:**
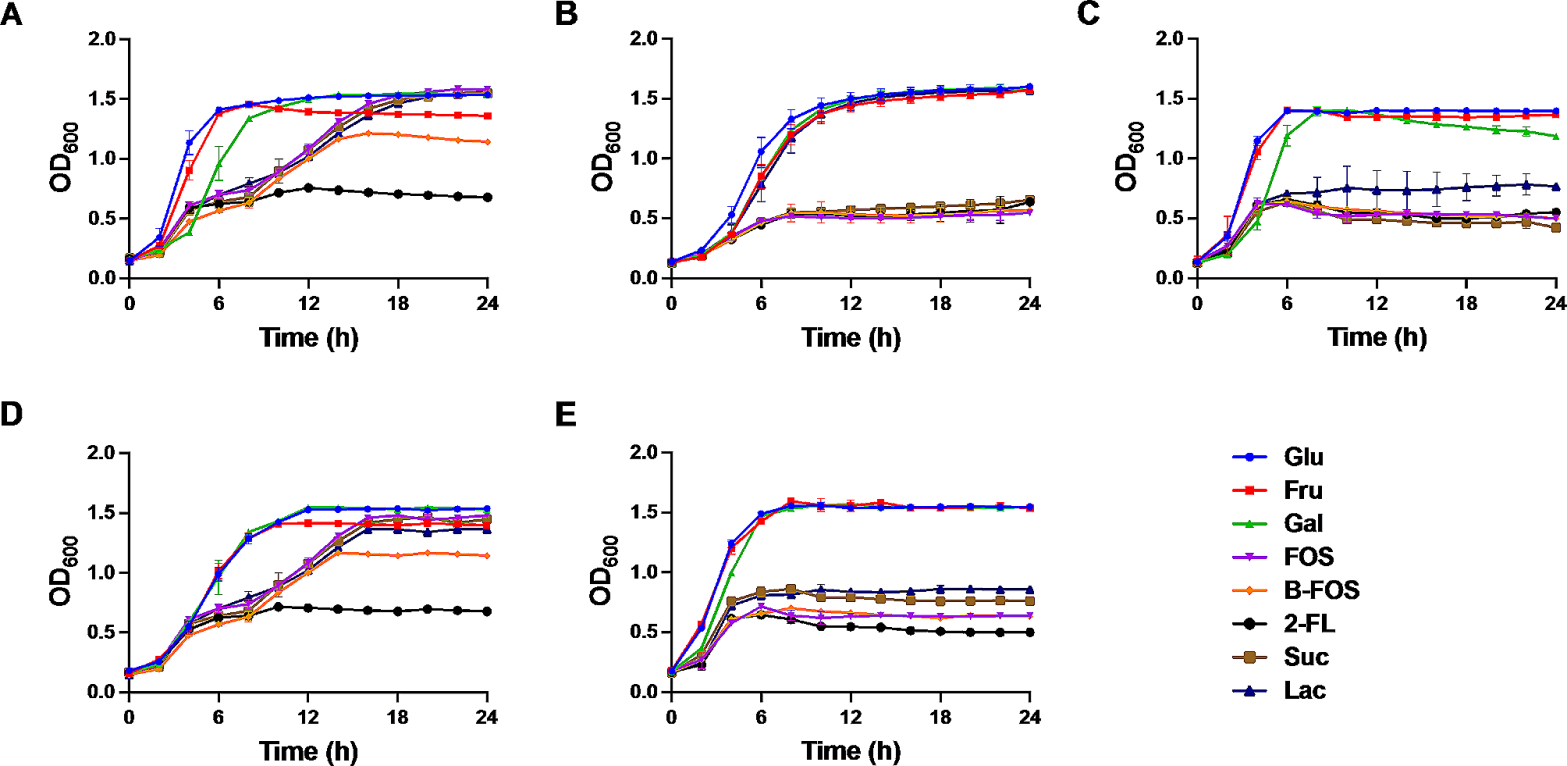
Growth curve of ethanol-tolerant LAB with different carbon sources. (**A**) *L. paracasei* Alc1, (**B**) *L. rhamnosus* AA, (**C**) *P. acidilactici* Alc3, (**D**) *L. paracasei* Alc4, (**E**) *P. acidilactici* Alc5. Glu, glucose; Fru, fructose; Gal, galactose; FOS, fructooligosaccharides; B-FOS, butyl-fructooligosaccharides; 2-FL, 2-fucosyllactose; Suc, sucrose; Lac, lactose

A strong ability for bacterial adhesion to intestinal mucosa can enhance host–bacterial interactions and aid in the promotion of immunomodulatory effects—a critical selection criterion for a potential probiotic [24]. The choice of carbon source can impact bacterial growth and/or adhesion [25, 26]; therefore, the following carbon sources were evaluated: glucose (used as control), fructose, galactose, fructooligosaccharide (FOS), butyl-fructooligosaccharide (B-FOS), 2-fucosyllactose (2-FL), sucrose, and lactose. Figure 4 shows that the addition of glucose, fructose, galactose, or lactose significantly stimulated the growth of *L. rhamnosus* AA and that the addition of glucose, fructose, or galactose promoted the growth of the remaining LAB strains. All seven tested carbon sources (except for 2-FL) stimulated the growth of *L. paracasei* Alc1 and *L. paracasei* Alc4; however, the growth rates were slowed when treated with FOS, sucrose, lactose, or B-FOS.

**Fig. 5:**
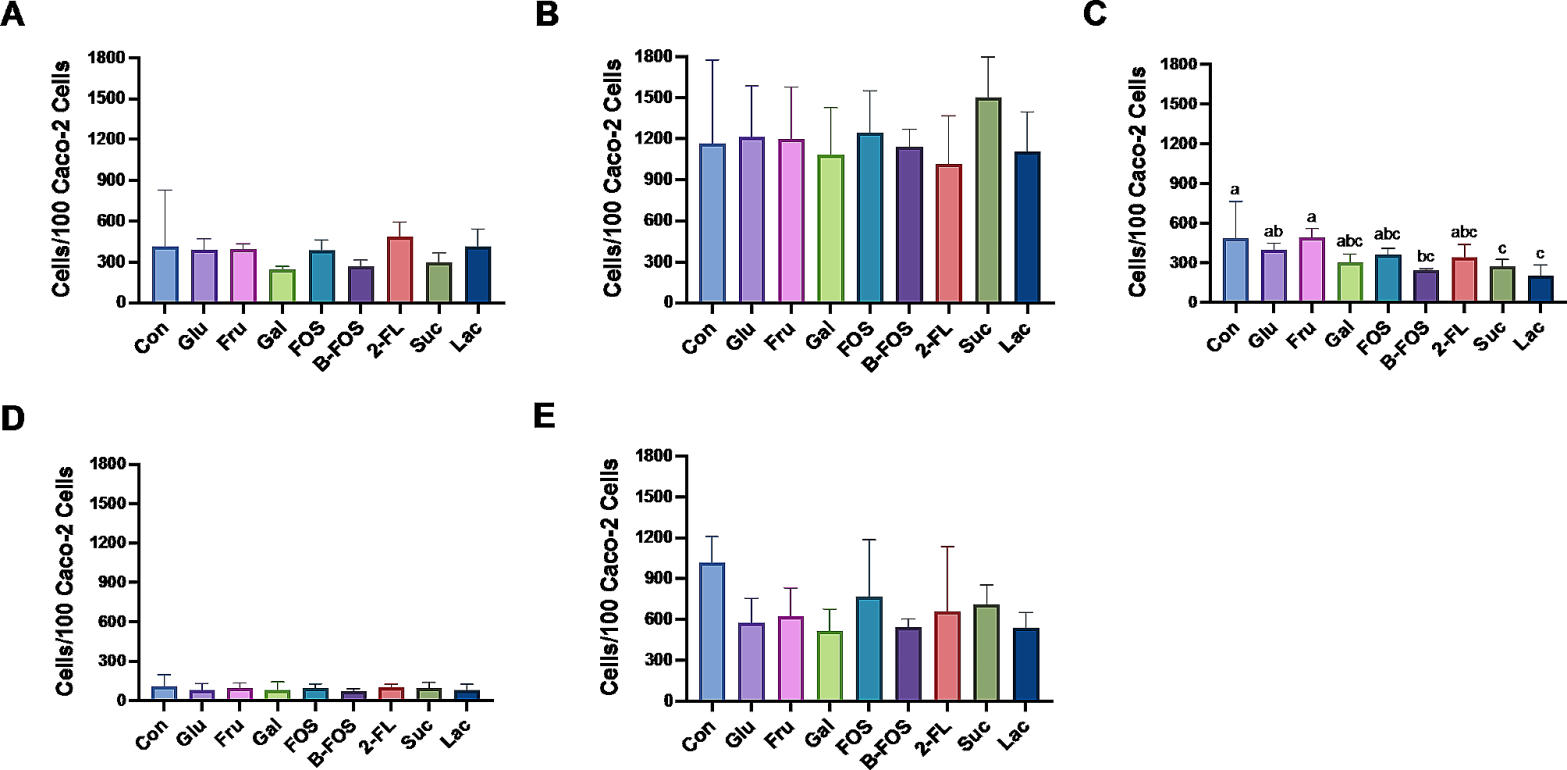
Adhesion ability of the selected LAB strains to Caco-2 cells monolayer with different carbon sources. (**A**) *L. paracasei* Alc1, (**B**) *L. rhamnosus* AA, (**C**) *P. acidilactici* Alc3, (**D**) *L. paracasei* Alc4, (**E**) *P. acidilactici* Alc5. Con, control; Glu, glucose; Fru, fructose; Gal, galactose; FOS, fructooligosaccharides; B-FOS, butyl-fructooligosaccharides; 2-FL, 2-fucosyllactose; Suc, sucrose; Lac, lactose. Treatments with different letters are significantly different at *p* < 0.05

The adhesion of the LAB strains to Caco-2 cells in response to different carbon sources was also investigated (Fig. 5). *L. rhamnosus* AA exhibited the strongest adhesion ability, whereas only few *L. paracasei* Alc4 was adhered to Caco-2 cells. Notably, the carbon sources had almost no effect on the adhesion abilities of the LAB strains (except for *P. acidilactici* Alc3), consistent with previous studies suggesting that most prebiotics have little impact on bacterial adhesion [27]. According to the classification of adhesion behaviors defined by Candela et al. [28], the adhesion ability of *L. rhamnosus* AA and *P. acidilactici* Alc5 are acceptable for probiotic applications.

**Fig. 6:**
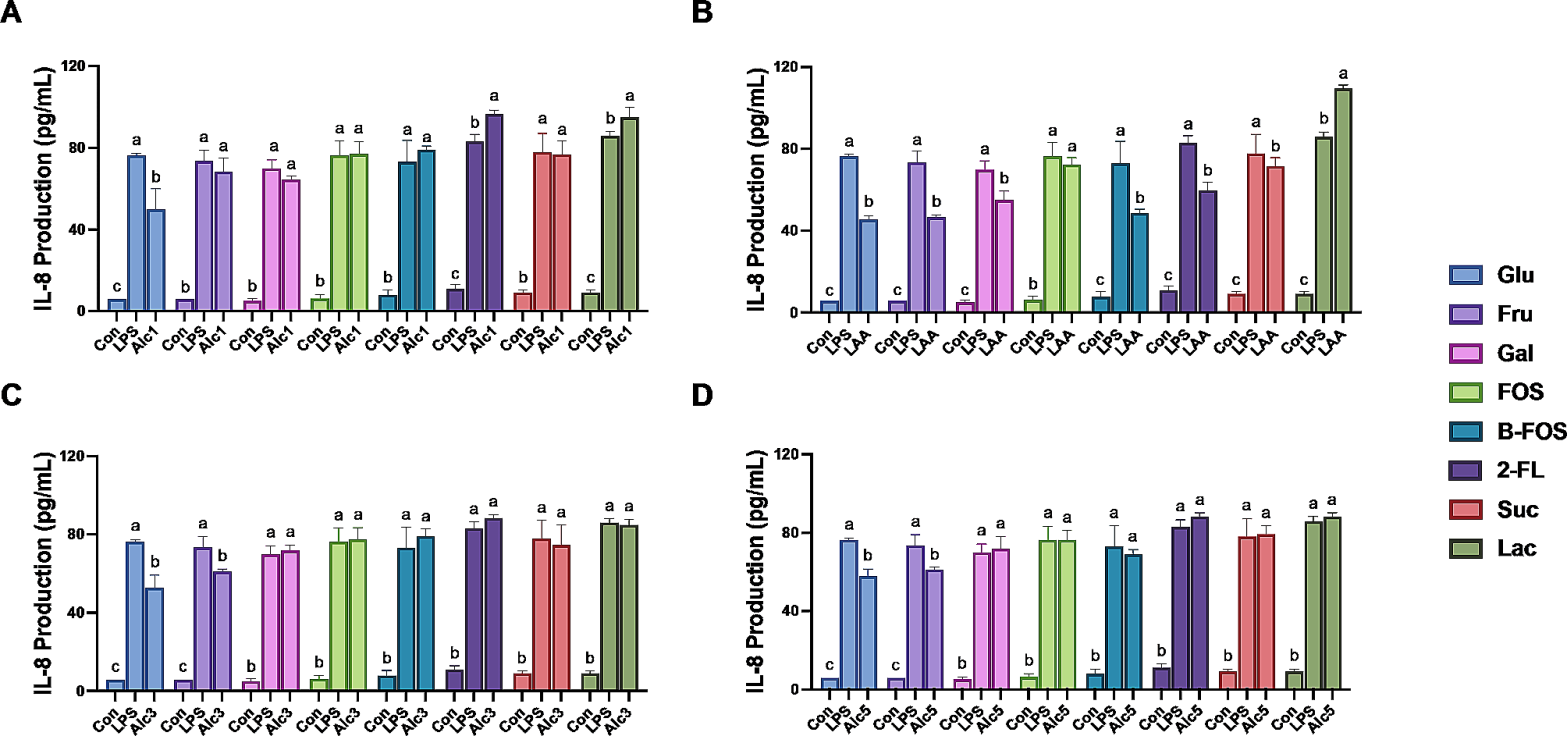
IL-8 production in lipopolysaccharide (LPS)-stimulated Caco-2 cells when co-cultured with *L. paracasei* Alc1 (**A**), *L. rhamnosus* AA (**B**), *P. acidilactici* Alc3 (**C**), *P. acidilactici* Alc5 (**D**) using different carbon sources. Con, control; LPS, LPS-treated cells; Glu, glucose; Fru, fructose; Gal, galactose; FOS, fructooligosaccharides; B-FOS, butyl-fructooligosaccharides; 2-FL, 2-fucosyllactose; Suc, sucrose; Lac, lactose. Treatments with different letters are significantly different at *p* < 0.05

**Fig. 7:**
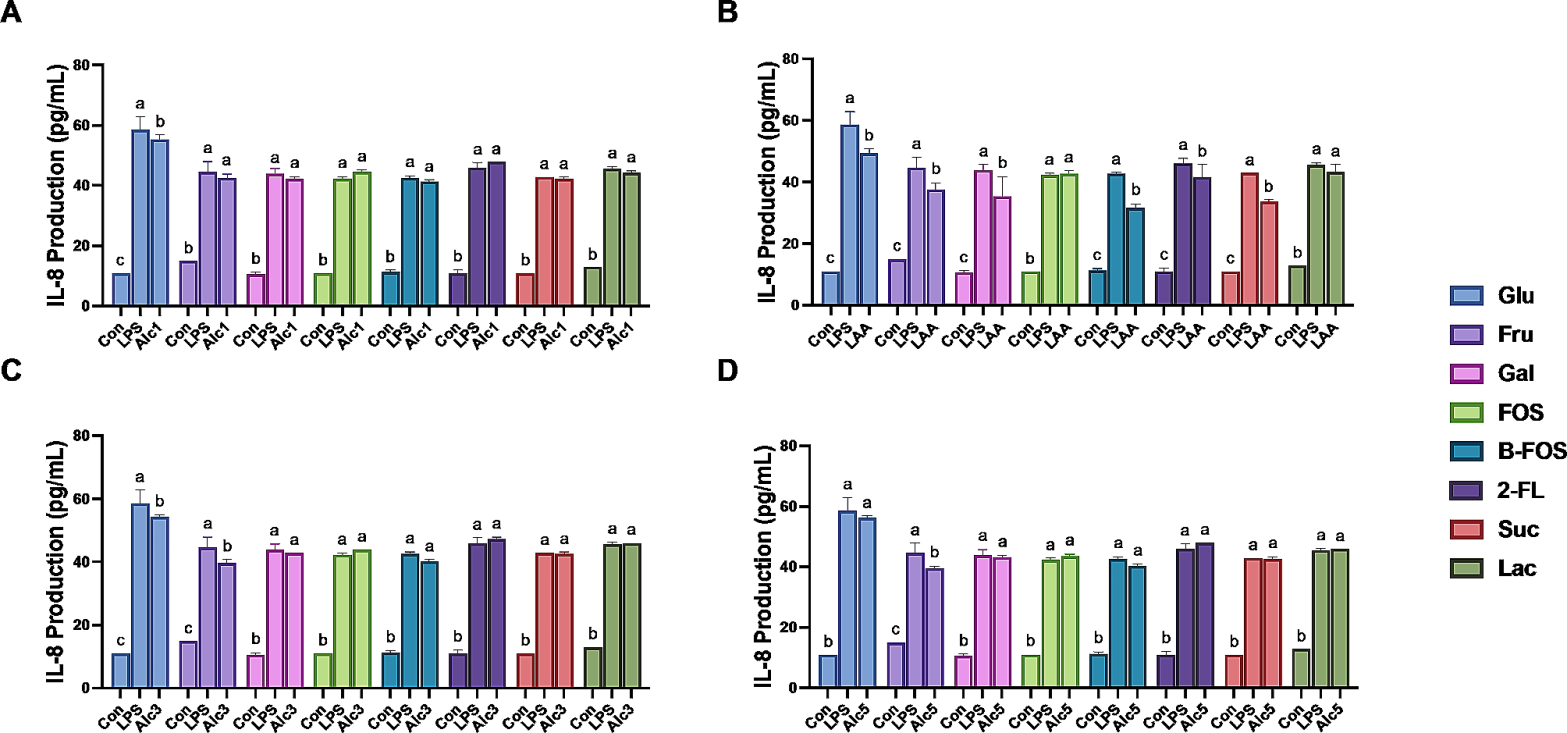
IL-8 production in lipopolysaccharide (LPS)-stimulated HT-29 cells when co-cultured with *L. paracasei* Alc1 (**A**), *L. rhamnosus* AA (**B**), *P. acidilactici* Alc3 (**C**), *P. acidilactici* Alc5 (**D**) using different carbon sources. Con, control; LPS, LPS-treated cells; Glu, glucose; Fru, fructose; Gal, galactose; FOS, fructooligosaccharides; B-FOS, butyl-fructooligosaccharides; 2-FL, 2-fucosyllactose; Suc, sucrose; Lac, lactose. Treatments with different letters are significantly different at *p* < 0.05

*L. paracasei* ALc4 showed a lack of adhesion ability and was not tested further. The remaining strains were utilized for the immunomodulatory assay in Caco-2 cell (Fig. 6) and HT-29 cell (Fig. 7) models. Both cell models indicated a similar tendency toward IL-8 alterations among the tested strains. Specifically, the LAA suspension significantly inhibited LPS-induced IL-8 secretion following the addition of glucose, fructose, galactose, B-FOS, 2-FL, or sucrose. *L. paracasei* Alc1, *P. acidilactici* Alc3, and *L. paracasei* Alc4 only suppressed IL-8 production when glucose or fructose was added.

### Genomic properties of *L. rhamnosus* AA

**Fig. 8:**
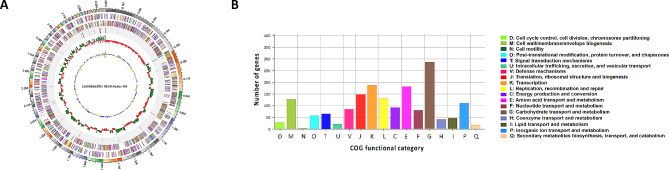
Circular genome map of *Lactobacillus rhamnosus* AA (**A**) and COG database annotation (**B**) based on whole genome sequencing. Five circles in the circular image indicates rRNA/tRNA, reverse CDS, forward CDS, GC ratio and GC skew from the outer periphery to the center

After exploring the ethanol tolerance, safety, adhesion ability, and immunomodulatory property of the five LAB candidates, we selected *L. rhamnosus* AA as a promising ethanol-tolerant probiotic. *L. rhamnosus* AA was subjected to whole genome sequencing to obtain its functional information. The whole genome size of *L. rhamnosus* AA was a 2,954,946 bp circular chromosome with an average GC content of 46.7% (Fig. 8A). The distribution of the Clusters of Orthologous Genes (COGs) displayed in Fig. 8B indicated that the most abundant COG categories were carbohydrate transport and metabolism (G), transcription (K), and amino acid transport and metabolism (E). Table 3 shows that the *adh* and *adhE* genes (encoding ADH and ALDH, respectively) were present in the genome, consistent with the ADH and ALDH activities detected in *L. rhamnosus* AA with or without sonication treatment (Fig. S1). Genes involved in glycolysis and gluconeogenesis are noted in Fig. S2.

**Table 3:** Predictive genes of *L. rhamnosus* AA related to alcohol dehydrogenase (ADH) and acetaldehyde dehydrogenase (ALDH)

Coding region	Length (aa)	EC	Product	NCBI gene
9210–10,034 (-)	825	1.1.1.2	Alcohol dehydrogenase (NADP (+))	*adh*
64,766–65,680 (+)	915	1.1.1.1	Alcohol dehydrogenase	*adh*
10,780–13,386 (+)	2607	1.2.1.10	Acetaldehyde dehydrogenase (acetylating)	*adhE*
15,661–16,683 (-)	1023	1.1.1.1	Alcohol dehydrogenase	*adh*
29,241–30,437 (+)	1197	1.1.1.-	Alcohol dehydrogenase YqhD	*adh2*

## Discussion

This study presents the selection and evaluation of LAB isolates with probiotic potential and resistance to alcoholic environments. Five ethanol-tolerant LAB isolates were screened from over 300 strains isolated from human feces. After assessment of the safety, adhesion ability, and immunomodulatory effects, the most promising alcohol-tolerant probiotic candidate, *L. rhamnosus* AA, was selected. Whole-genome analysis was performed to further confirm its probiotic potential, metabolic pathways, and the presence of ADH and ALDH at genomic level.

A previous pilot study demonstrated that an eight week supplementation with *Lactobacillus acidophilus* and *Bifidobacterium lactis* in healthy adults enhanced the counts of supplement-specific probiotics in their intestines but had no effect on alcohol metabolism following acute alcohol drinking [29]. Another randomized controlled trial reported that intake of milk fermented with *L. rhamnosus* GG (with ALDH activity) prior to alcohol ingestion reduced blood and salivary acetaldehyde levels [30]. Notably, this reduction was more significant in individuals displaying alcohol flushing than in non-flushing people [30]. Thus, the results vary with the probiotic bacterial strains, indicating that selection of probiotic strain(s) with high ethanol- and acetaldehyde-metabolizing abilities is essential. Other studies have attempted to increase alcohol degradation in the intestine after oral administration by designing recombinant bacterial strains, such as *Lactococcus lactis* expressing human ADH1B [31], *Bacillus subtilis* co-expressing ADH and ALDH [32], and *Escherichia coli* Nissle 1917 co-expressing ADH and ALDH [33]. However, the safety of these recombinant probiotics for human use remains uncertain. To the best of our knowledge, our study is the first to report the whole genome sequence of a screened ethanol-tolerant LAB strain that shows both ADH and ALDH activities. Future studies will focus on the impact of *L. rhamnosus* AA on alcohol degradation and the detoxification of alcohol-derived acetaldehyde in animal models and clinical trials.

The popular consumption of alcoholic beverages throughout the world has brought about increasing global health problems [34]. Frequent alcohol consumption is considered as a major cause of liver damage, which may further develop into alcoholic fatty liver, hepatic fibrosis, and inflammatory hepatitis [1, 35]. At the same time, alcohol abuse disrupts the gut microbiota and triggers mucosal inflammation and pathogenic attack, further exacerbating the alcohol-induced hepatic injuries [36]. Approximately 36–45% of the East Asian population experience facial redness following alcohol intake (termed Asian Alcohol Flushing Syndrome). These individuals have a genetic deficiency of ALDH, and the excessive accumulation of acetaldehyde causes their alcohol flushing response [4, 37]. Generally, the normal human gut microbiota is capable of converting ethanol into acetaldehyde via ADH but the gut bacteria have low ALDH activities, leading to high levels of acetaldehyde in the large intestine [38]. Compared to the non-flushers, people with the alcohol flushing response are more vulnerable to the risks of alcohol consumption even with a low alcohol intake [37].

Consumer needs and desires are now propelling the beverage industry in new directions, with increasing demand for healthy and functional products [39]. Thus, the development of alcoholic beverages enriched with functional probiotics capable of producing enzymes like ADH and ALDH could open up new markets and/or create distinct product categories [40]. Among the functional foods and beverages with health benefits, those containing probiotics have stood out in the marketplace for decades [41]. Probiotic properties include gut microbiota modulation, immune system simulation, and protection against infections [42, 43]. Previous studies have suggested that red ginseng and pear juice reduce hangover symptoms by decreasing acetaldehyde formation [44, 45], whereas probiotic interventions could potentially promote the detoxification of acetaldehyde [30]. Some LAB strains have been reported for their ADH and/or ALDH activities that enable them to reduce alcoholic toxicity [46]. For instance, *Lactobacillus rhamnosus* GG is a commercial probiotic strain that shows a limited ability to degrade ethanol but presents an appreciable in vitro acetaldehyde-metabolizing capacity [30, 47].

Despite this potential, the growth and metabolism of LAB strains strongly depend on external environmental factors (i.e., ethanol concentration) [48]. Currently, the few alcoholic beverages that have been supplemented with LAB are mild alcoholic products, such as omegisool (a Korean corn-fermented beverage) and lugri (a rice-fermented beverage in the Himalayas). These beverages contain *Lactobacillus* and *Pediococcus*, and their alcohol percentages are less than 2.0% (v/v) and 0.56% (v/v), respectively [49, 50]. Thus, optimizing the selection of probiotics with alcohol resistance and functional properties in health remains challenging [36].

## Conclusion

The LAB strain *L. rhamnosus* AA was screened and selected as a promising ethanol-tolerant probiotic strain that can survive under ethanol stress and adhere to the intestinal epithelial cells to induce their anti-inflammatory effects. The genome of this strain contains *adh* and *adhE* genes for ADH and ALDH production for alcohol metabolism. Use of this strain as a probiotic could help to reduce the physical burden after alcohol consumption, especially in individuals with the alcohol flushing response. In the future, *L. rhamnosus* AA could be potentially added to beverages to create functional alcoholic drinks.

### Electronic supplementary material

Below is the link to the electronic supplementary material.

Supplementary Material 1

## Data Availability

No datasets were generated or analysed during the current study.
