# Supplementary material for: Investigating human-derived lactic acid bacteria for alcohol resistance
Source: Microb Cell Fact. 2024 Apr 24;23:118. doi: 10.1186/s12934-024-02375-4 (PMC11040769; doi:10.1186/s12934-024-02375-4)
Supplement: Supplementary file 1 — Supplementary Material 1 [file 12934_2024_2375_MOESM1_ESM.docx]

**Supplementary materials**

**Fig. S1.** Activities of alcohol dehydrogenase (ADH) and acetaldehyde dehydrogenase (ALDH) in *L. rhamnosus* AA


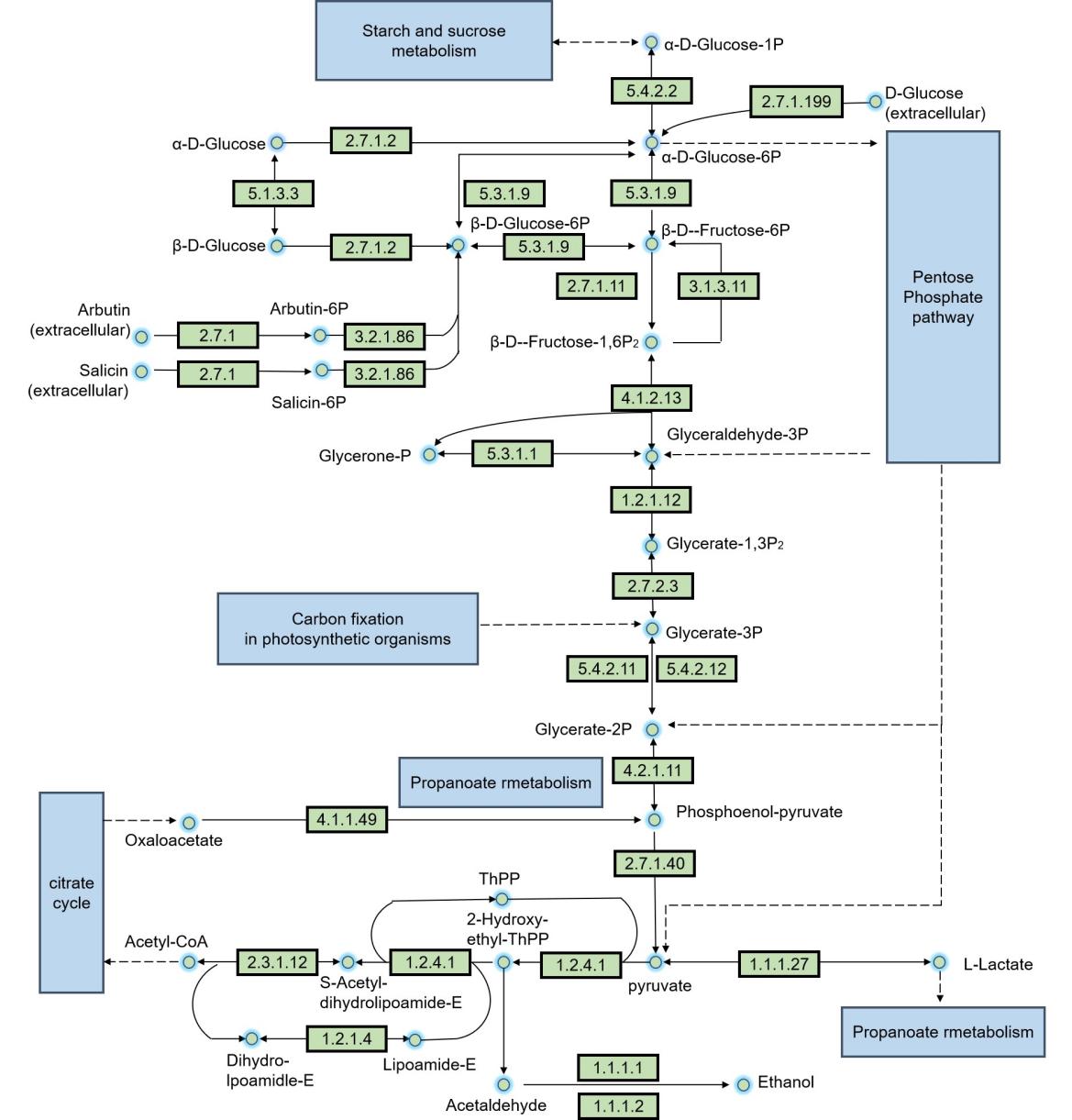


**Fig. S2.** Glycolysis and gluconeogenesis pathway.

Green color-filled boxes indicated the genes detected in the genome of *L. rhamnosus* AA.
